# Duration between aneurysm rupture and treatment and its association with outcome in aneurysmal subarachnoid haemorrhage

**DOI:** 10.1038/s41598-022-27177-9

**Published:** 2023-01-27

**Authors:** Isabel C. Hostettler, Nicole Lange, Nina Schwendinger, Samira Frangoulis, Theresa Hirle, Dominik Trost, Jens Gempt, Kornelia Kreiser, Maria Wostrack, Bernhard Meyer

**Affiliations:** 1 Department of Neurosurgery, Klinikum Rechts der Isar, Technical University Munich, Ismaningerstrasse 22, 81675 Munich, Germany; 2 Department of Neurosurgery, Cantonal Hospital St. Gallen, St. Gallen, Switzerland; 3 Department of Neuroradiology, Klinikum Rechts der Isar, Technical University Munich, Munich, Germany

**Keywords:** Neuroscience, Neurology

## Abstract

Timely treatment of aneurysmal subarachnoid haemorrhage (aSAH) is key to prevent further rupture and poor outcome. We evaluated complications and outcome adjusting for time from haemorrhage to treatment. Retrospective analysis of aSAH patients admitted between 2006 and 2020. Data was collected using standardized case report forms. We compared risk factors using multivariable logistic regression. We included 853 patients, 698 (81.8%) were treated within 24 h. Patients with higher Hunt and Hess grades were admitted and treated significantly faster than those with lower grades (overall p-value < 0.001). Fifteen patients (1.8%) rebled before intervention. In the multivariable logistic analysis adjusting for timing, Barrow Neurological Institute score and intracerebral haemorrhage were significantly associated with rebleeding (overall p-value 0.006; OR 3.12, 95%CI 1.09–8.92, p = 0.03, respectively) but timing was not. Treatment > 24 h was associated with higher mortality and cerebral infarction in only the subgroup of lower grades aSAH (OR 3.13, 1.02–9.58 95%CI, p-value = 0.05; OR 7.69, 2.44–25.00, p-value < 0.001, respectively). Therefore treatment > 24 h after rupture is associated with higher mortality and cerebral infarction rates in lower grades aSAH. Delay in treatment primarily affects lower grade aSAH patients. Patients with lower grade aSAH ought to be treated with the same urgency as higher-grade aSAH.

## Introduction

Subarachnoid haemorrhages make up 5% of all strokes^1^. Despite improvements in risk stratification, imaging, surgical and intensive care treatment, productive life-years are significantly reduced due to a mortality of up to 50% and surviving patients exhibiting permanent neurological and cognitive deficits allowing only 6–17% of survivors to return to work^2–5^.

Early treatment (first 24–48 h) can prevent rebleeding and consecutively reduce likelihood for poor functional outcome^6,7^. Several factors influence treatment delay, most importantly delay in hospital admission and delay in treatment. Delay in hospital admission is most frequently caused by the need to transfer a patient from another hospital. This can be due to initial misdiagnosis, reported to be as high as 51%^8–10^. Delay in correct diagnosis has markedly decreased as shown in previous studies^11^. On the other hand, it can be due to delayed presentation of a patient to hospital in case of misjudgement of initially mild symptoms. Patients with delay in aneurysm occlusion are at increased risk for complications, such as rebleeding, cerebral vasospasm, cerebral infarct and poor functional outcome^12–14^. Another important factor causing treatment delay is the actual delay due to the standard of a specific clinic. A previous study demonstrated patients with delayed admission to be at increased risk for cerebral infarcts^15^. Still no clear guidelines exist on timing of ruptured aneurysm treatment although several studies advocate early treatment (first 1–3 days)^15–17^. A study showed advantages for ultra-early (within the first 24 h) aneurysm treatment^16^. However, this study dichotomized patients into treated within 24 and after 48 h excluding patients who were treated in-between.

We aim to evaluate the association of time from acute haemorrhage to aneurysm treatment in a large cohort of patients with ruptured intracranial aneurysms (IA). We hypothesize that delay in treatment has an influence on several outcome parameters such as rebleeding, cerebral infarction, functional outcome on discharge, and mortality.

## Patients and methods

### Population

**Figure 1:**
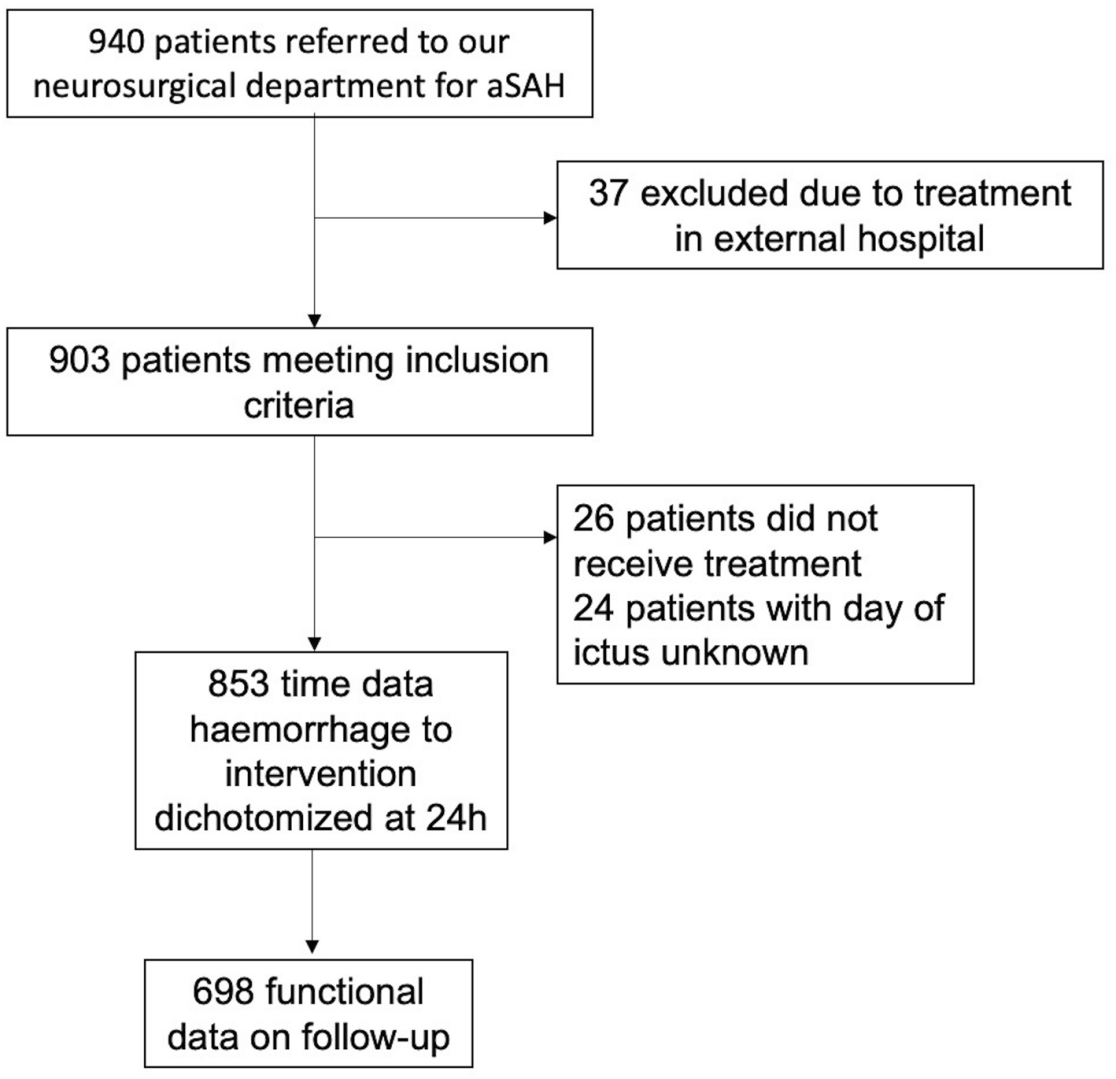
Study flow chart.

We prospectively recruited patients with aSAH into our hospital-based registry and conducted a retrospective analysis in patients with aSAH treated between March 2006 and March 2020. We did not consider patients with unruptured IA, non-aneurysmal SAH and patients suffering from an aSAH due to a different underlying pathology (e.g. mycotic aneurysms). For our main analysis we only included patients who had known time of ictus and time of treatment, therefore time from haemorrhage to intervention could be assessed (Fig. 1). We excluded patients who died before their aneurysm could be treated as influence of timing to treatment cannot be evaluated in these patients.

We collected information on patient demographics, risk factors such as smoking, and other medical history from patient self-reporting and, if not possible, from hospital medical records. Our outcomes of interest were rebleeding, cerebral infarct, functional outcome on discharge as well as mortality. We measured functional outcome on discharge using the Glasgow Outcome Scale (GOS), dichotomizing it into 1–3 for unfavourable and 4–5 for favourable functional outcome^18^.

The main independent variables of interest were time from haemorrhage to treatment and time from admission to treatment of the ruptured aneurysm. Time of ictus was set to when patients reported first onset of severe headaches (typically thunderclap headache), they had witnessed loss of consciousness, seizure or onset of other neurological deficits. We dichotomized timing from ictus to treatment into before or after 24 h and will refer to it from here on with “treatment < / > 24 h”. We conducted a subgroup analysis additionally stratifying patients who were admitted within 24 h into following subgroups if detailed time from haemorrhage to admission and therefore treatment was available: < 4 h, < 6 h, and < 12 h, respectively.

### Radiological data

Rebleeding was defined as a bleeding which occurred from the previously established ruptured aneurysm. We only considered rebleedings before treatment of the ruptured aneurysm which were proven by imaging. Cerebral infarct was defined as newly developed, radiologically proven infarct occurring beyond 48 h of intervention^19^. We assessed the Barrow Neurological Institute (BNI) scale as previously described^20^. It consists of grade 1–5 depending on the measured SAH thickness. The BNI scale was the only variable with missing data as it was only assessed if initial CT scan was available; a complete case analysis was performed in this case.

### Statistical analysis

We present categorical variables as count and percentage; continuous variables as mean with standard deviation (SD) in case of normal distribution or median with interquartile range (IQR) in case of non-normal distribution.

We first conducted a univariable analysis. For the multivariable logistic regression analysis, we adjusted the model with the prespecified variables age and time from haemorrhage to intervention as well as variables with a p-value of ≤ 0.1. We conducted one main and a subgroup analysis: the main analysis was based on patients who could be dichotomized into treatment within or after 24 h from ictus. This analysis was repeated by Hunt and Hess (HH) grade (1/2, 3, 4/5) to evaluate for difference based on aSAH grade. Finally, the subgroup analysis was conducted for patients for whom detailed time from the haemorrhage to treatment was available. The level of statistical significance was set at 5% (p-value = 0.05).

Statistical analysis was performed using STATA 15 (StataCorp. 2011. *Stata Statistical Software: Release 15*. College Station, TX: StataCorp LP).

### Ethical approval

The study was approved by the local Research Ethics committee of the Technical University of Munich (186/20S). As this was a retrospective analysis and none of the included data harbours the possibility of being tracked back to a specific patient, the Research Ethics committee of the Technical University of Munich deemed our study exempt for retrospective informed consent for the included patients. All methods were performed in accordance with the relevant guidelines and regulations.

## Results

Of 940 patients referred to our department we included 853 patients with acute aSAH and available information on timing from haemorrhage to intervention in days stratifying them into treatment < 24 h or > 24 h (Fig. 1). For 535 patient timing from haemorrhage to intervention was available in hours while timing for the rest could only be dichotomized into > and < 24 h. Baseline characteristics are summarized in Table 1. Most patients were female (66.6%), mean age was 57.3 years (14.3 SD). The majority of patients were treated with coiling while 312 patients (36.6%) were treated via clipping. Overall, 31.2% had a Hunt and Hess (HH) grade of 4 or 5. In-hospital mortality rate was 13.8%. 72.8% had first to be transferred from a primary or secondary care centre, with only 27.2% being directly admitted to our hospital.

**Table 1:** Baseline characteristics.

	All N = 853	Treatment < 24 h, n = 698	Treatment > 24 h, n = 155	p-value
Age, mean (SD)	57.3 (14.3)	57.3 (14.1)	57.3 (15)	0.98
Female sex, N (%)	568 (66.6)	470 (67.3)	98 (63.2)	**0.33**
Smoker	105 (12.3)	85 (12.2)	20 (12.9)	0.8
**Hunt and Hess, N (%)**				< 0.001
1	116 (13.6)	68(9.7)	48 (31)	
2	286 (33.5)	215 (30.8)	71 (45.8)	
3	185 (21.7)	164 (23.5)	21 (13.6)	
4	152 (17.8)	139 (19.9)	13 (8.4)	
5	114 (13.4)	112 (16.1)	2 (1.3)	
**BNI score**	803/853	674/698	129/155	< 0.001
1	40 (5.0)	26 (3.9)	14 (10.9)	
2	218 (27.2)	166 (24.6)	52 (40.3)	
3	377 (46.9)	334 (49.6)	43 (33.3)	
4	132 (16.4)	116 (17.2)	16 (12.4)	
5	36 (4.5)	32 (4.8)	4 (3.1)	
Associated ICH	200 (24.5)	168 (24.6)	32 (24.1)	0.90
Associated IVH	419 (51.4)	382 (56.0)	37 (28.8)	< 0.001
Clipping, N (%)	314 (36.8)	263 (37.7)	49 (31.6)	**0.16**
**Complications, N (%)**				
Rebleeding	15 (1.8)	13 (1.9)	2 (1.3)	0.63
CVS	489 (57.3)	406 (58.2)	83 (53.6)	**0.29**
Infarction	163 (19.1)	139 (19.9)	24 (15.5)	**0.21**
Decompressive craniectomy	122 (14.3)	116 (16.6)	6 (3.9)	< 0.001
VPS	211 (24.7)	192 (27.5)	19 (12.3)	< 0.001
In-hospital mortality	118 (13.8)	103 (15.5)	15 (9.7)	**0.1**
**GOS at discharge, N (%)**				< 0.001
1	118 (13.8)	103 (14.8)	15 (9.7)	
2	103 (12.1)	94 (13.5)	9 (5.8)	
3	137 (16.1)	121 (17.3)	16 (10.3)	
4	140 (16.4)	121 (17.3)	19 (12.3)	
5	355 (41.6)	259 (37.1)	96 (61.9)	

*BNI* Barrow Neurological Institute, *CVS* cerebral vasospasm,* GOS* Glasgow Outcome Scale,* ICH* intracerebral haemorrhage,* IVH* intraventricular haemorrhage,* SD* standard deviation,* VPS* ventriculoperitoneal shunt.

Significant values are in bold.

### Time from haemorrhage and admission to intervention, associated complications

We evaluated time from haemorrhage and admission to aneurysm treatment, respectively (Table 2). Patients with a higher HH grade were admitted and treated significantly faster than those with lower grades (overall p-value < 0.001). Patients who received treatment < 24 h more frequently underwent decompressive craniectomy (16.6% vs. 3.9%). Patients who underwent clipping experienced a significant delay from admission to treatment (coefficient 14.51, 5.00–24.03 95%CI, p = 0.003): overall median time from admission to treatment was 9.7 h for clipping and 5.5 h for coiling.

**Table 2:** Timing to treatment divided by HH grades and treatment.

	Coiling	Clipping
**Time haemorrhage to treatment (hours)**		
HH ½	19.5	25.0
HH 3	14.5	15.7
HH 4	11.8	11.5
HH 5	7.8	7.7
**Time admission to treatment (hours)**		
HH ½	6.3	12
HH 3	6.8	10
HH 4	6	7.3
HH 5	3.5	4.3

*HH* Hunt and Hess.

### Rebleeding

Overall, 15 patients (1.8%) in our cohort rebled before the ruptured aneurysm was occluded (Table 3). Mean age was 60.9 years (SD 14.8) and 9 (69.2%) were female. None of them had HH 1, six patients had HH5 and 3 had HH 2, 3 and 4 each. Eight were treated via clipping (53.3%) and 7 (46.7%) coiling. Seven patients (46.7%) rebled within 24 h. Delay in treatment was usually caused by transfer from another hospital.

**Table 3:** Multivariable analysis rebleeding.

	OR	95% CI	P value
Treatment > 24 h	0.80	0.17–3.71	0.77
**BNI score**			**0.006**
- 1	None		
- 2	0.20	0.05–0.80	
- 3	0.11	0.03–0.42	
- 4	0.05	0.01–0.50	
- 5 (reference group)			
ICH	3.12	1.09–8.92	**0.03**

*BNI* Barrow Neurological Institute, *ICH* intracerebral haemorrhage.

Significant values are in bold.

None of the patients treated within 4 h from haemorrhage rebled. In the multivariable regression analysis (Table 3), following factors were independently associated with rebleeding: ICH (OR 3.12, 1.09–8.92 95%CI, p = 0.03) as well as BNI score (overall p-value 0.006). Treatment > 24 h was not associated with rebleeding before aneurysm occlusion (OR 0.80, 0.17–3.71 95%CI, p = 0.77).

In a subgroup analysis stratifying into ultra-early treatment within < 4 h, < 6 h, and < 12 h respectively, treatment was not associated with rebleeding (p-value 0.23, 0.84 and 0.33, respectively in the multivariable analysis).

### Cerebral infarct

163 (19.1%) developed cerebral infarcts. In the multivariable logistic analysis (Table 4) only HH grade on admission remained significantly associated with cerebral infarct (overall p-value < 0.001). BNI score and ICH showed some evidence although they were not statistically significant. Hunt and Hess showed gradual increase of OR by subgroup. Treatment > 24 h was not associated with cerebral infarct in the overall cohort (OR 1.56, 0.98–2.70 95%CI, p = 0.1). In the subgroup analysis of stratification into < 4 h, < 6 h, and < 12 h, treatment was not associated with cerebral infarct development (p-values 0.7, 0.86 and 0.62, respectively).

**Table 4:** Multivariable analysis cerebral infarct.

	OR	95% CI	P value
Treatment > 24 h	1.56	0.92–2.70	0.1
Age	0.99	0.98–1.0	0.1
**BNI score**			0.06
- 1 (reference group)			
- 2	1.15	0.43–3.08	
- 3	1.9	0.74–4.89	
- 4	1.19	0.43–3.28	
- 5	2.74	0.85–8.89	
**HH**			** < 0.001**
- 1 (reference group)			
- 2	1.73	0.76–3.94	
- 3	2.63	1.11–6.22	
- 4	4.57	1.92–10.91	
- 5	6.43	2.64–15.66	
ICH	1.45	0.97–2.18	0.07
IVH	0.96	0.64–1.42	0.82

*BNI* Barrow Neurological Institute, *HH* Hunt and Hess, *ICH* intracerebral haemorrhage, *IVH* intraventricular haemorrhage.

Significant values are in bold.

In a subgroup analysis, patients with lower grades aSAH (HH 1–3) treated > 24 h had a higher probability of cerebral infarction compared to those with earlier treatment (OR 1.85, 1.01–3.33, p-value = 0.05). This was even more pronounced in HH grade 3 patients (OR 7.69, 2.44–25.00 95%CI, p-value < 0.001) in the multivariable regression analysis adjusting for age, BNI score and ICH.

### Mortality

In the multivariable logistic regression analysis, age and HH grade remained significantly associated with increased in-hospital mortality (OR 1.04, 95% CI 1.02–1.05, p < 0.001; overall p-value < 0.001, respectively; Table 5). Treatment > 24 h was not associated with higher in-hospital mortality in the multivariable analysis.

**Table 5:** Multivariable analysis in-hospital mortality.

	OR	95% CI	P value
Treatment > 24 h	1.56	0.80–3.03	0.19
Age	1.04	1.02–1.05	** < 0.001**
**BNI score**			0.64
- 1 (reference group)			
- 2	0.72	0.23–2.27	
- 3	1.13	0.38–3.33	
- 4	1.05	0.34–3.29	
- 5	0.8	0.2–3.22	
**HH grade**			** < 0.001**
- 1 (reference group)			
- 2	1.6	0.44–5.82	
- 3	3.83	1.08–13.68	
- 4	6.07	1.7–21.73	
- 5	17.97	5.0–64.64	
ICH	1.21	0.75–1.94	0.44
IVH	1.22	0.75–1.98	0.42

*BNI* Barrow Neurological Institute, *HH* Hunt and Hess, *ICH* intracerebral haemorrhage, *IVH* intraventricular haemorrhage.

Significant values are in bold.

In the subgroup analysis stratifying into < 4 h, < 6 h, and < 12 h, none of these time frames were significantly associated with a lower mortality rate although treatment < 4 h showed some evidence for higher mortality (OR 2.27, 0.96–5.35 95%CI, p = 0.06 in the multivariable analysis) most probably due to an increased rate of higher-graded SAH within this ultra-early time frame group as shown above.

In a subgroup analysis looking at HH1/2, HH3 and HH4/5 separately, patients with HH grade 3 treated > 24 h had a higher likelihood for in-hospital mortality (OR 3.13, 95% CI 1.02–9.58, p-value = 0.05) in the multivariable regression analysis adjusting for age, ICH and IVH. None of the patients with HH grades 1 and 2 treated within 24 h died.

### Functional outcome at discharge

Treatment > 24 h did not exhibit any statistically significant association with poor GOS at discharge in the multivariable analysis; however, age (OR 1.05, 95%CI 1.04–1.07, p-value < 0.001), HH grade (overall p-value < 0.001), IVH (OR 264, 95%CI 1.7–4.09, p-value < 0.001) and smoking (OR 1.69, 95%CI 1.16–2.47, p = 0.007) were still associated (Table 6). All patients admitted with HH grade 5, except for two, had an unfavourable GOS at discharge.

**Table 6:** Multivariable analysis dichotomized GOS at discharge.

	OR	95% CI	P value
Treatment > 24 h	1.16	0.68–2.04	0.58
Age	1.05	1.04–1.07	** < 0.001**
**BNI score**			0.67
- 1 (reference group)			
- 2	0.74	0.28–1.96	
- 3	0.79	0.31–2.05	
- 4	0.75	0.27–2.08	
- 5	1.49	0.41–5.36	
**HH**			** < 0.001**
- 1 (reference group)			
- 2	1.39	0.65–2.95	
- 3	4.52	2.1–9.72	
- 4	15.1	6.72–33.96	
- 5	50.08	19.86–126.26	
ICH	1.04	0.7–1.56	0.84
IVH	2.64	1.7–4.09	** < 0.001**
Smoking	1.69	1.16–2.47	**0.007**

*BNI* Barrow Neurological Institute, *HH* Hunt and Hess, *ICH* intracerebral haemorrhage, *IVH* intraventricular haemorrhage.

In the subgroup analysis of timing stratification into < 4 h, < 6 h, and < 12 h no relevant associations were found (p-value 1, 0.81 and 0.78, respectively).

## Discussion

In our cohort, treatment > 24 h after aneurysm rupture was associated with a higher mortality and cerebral infarction rate in lower grade aSAH patients. Patients with lower grade aSAH but timely treatment had a lower likelihood for cerebral infarct and in-hospital mortality. The higher the HH grade the sooner patients were admitted and the ruptured aneurysm was treated.

Ruptured IA are commonly treated within 24–48 h after haemorrhage to reduce the risk of rebleeding and therefore complications^6,7,21^. In line with previous data our data shows that early treatment in patients with aSAH is safe and influences outcome in a positive way^14^. In our department, patients suffering from aSAH are treated via coiling or clipping within a median of 7 h of the haemorrhage: aSAH patients are treated as emergency; they do not have to wait until the next elective slot. In over 70% patients with a delay in treatment they were transferred from another hospital explaining this delay at least in part. Another important and well known factor in delay in lower grades aSAH is misdiagnosis (by the patient or even the clinician)^8–10^. In our cohort however, complications rates were low. Reported rebleeding rates differ significantly in the literature reaching up to 27%, however decreasing in recent years^6,22–24^. Rebleeding rate in our cohort was lower. This likely reflects the fact that patients admitted to our neurosurgical department are usually treated within a median of 7 h from admission (Table 2) preventing rebleeding in many cases. We did find an association of early aneurysm treatment and decreased rebleeding risk (although this association could not be confirmed in our multivariable analysis due to the low number of rebleedings): none of the patients treated within 4 h rebled. We believe that the percentage of rebleedings in our cohort reflects the current actual rate more adequately than the currently in the literature available rebleeding rates.

Some people might question why higher grade aSAH patients in our cohort were treated earlier than lower grade aSAH patients. Our hypothesis is that most of it comes down to treatment delay. Patients with initial lower grade aSAH were primarily affected by treatment delay. We do believe that this is the cause for consequently higher complication rates in lower grades aSAH. A previous study showed that early surgery (within 48 h) was associated with better outcome^14^. Although they only included patients undergoing clipping and WFNS grades 1–3 and therefore cannot draw any conclusions on higher aSAH grades, this underlines the findings in our cohort in lower aSAH grades. Regardless of initially mild symptoms and commonly assumed stable clinical state, treatment delay should be avoided, and treatment pursued with the same urgency as in higher-grade aSAH patients. We therefore emphasise aneurysm occlusion within 24 h regardless of the HH grade.

In line with our data, previous studies report that patients who are admitted sooner have worse functional outcomes^15^. Our higher mortality rate when patients are treated < 4 h more likely reflects the severity of the haemorrhage rather than a real association of treatment timing and adverse events. However, exact timing was only available in a subgroup of patients (62.7%).

The observations that patients with HH grade 4 and 5 treated < 6 h had a higher mortality rate reflects haemorrhage severity rather than patients with early treatment having worse functional outcome. The poorer the clinical state of a patient the quicker the transfer and consequently earlier the admission to a maximum care facility. It is not surprising that patients with lower aSAH grades and hence minor symptoms present with a delay after the haemorrhage, commonly due to persisting or increasing headaches and therefore have fewer complications and generally a more favourable functional outcome. Hunt and Hess grades 1 and 2 are commonly considered less severe and somewhat more stable with a decreased complication risk. Our findings that patients with a lower grade aSAH treated > 24 h from haemorrhage have a higher likelihood of in-hospital mortality and cerebral infarction indicates that although these HH grades might be considered as more favourable, time from haemorrhage to treatment significantly influences functional outcome in a patient group that has even more to lose. Due to the urgency high grade aSAH patients are handled with compared to lower grade aSAH patients, the negative influence of timing only becomes apparent when the delay occurs. A rapid and safe occlusion of the ruptured aneurysm is therefore as important in lower grade aSAH as it is in higher grades and treatment should not be delayed.

Controversy about outcome after clipping compared to coiling remains. The previously published CARAT study reported low rates of aneurysm rupture after both surgical or endovascular treatment^25^. With regards to outcome, several studies report worse functional outcome in patients undergoing clipping compared to coiling^26–28^. We believe this finding most likely reflects treatment delay in patients undergoing clipping occurring due to several reasons with our data supporting this hypothesis: in line with other studies patients undergoing clipping had a significantly longer time from admission to treatment than did patients undergoing coiling^29^. However, despite the treatment delay in patients undergoing clipping, this did not reflect in a higher rebleeding rate nor higher rate of unfavourable outcome. This might indicate that despite the potentially higher periprocedural morbidity of clipping, it might still be as safe as coiling, in line with previous studies^30,31^. Another hypothesis might be that patients who were clipped have worse HH grades. We did not find a significant difference in mortality rate and unfavourable outcome on discharge comparing clipped versus coiled patients.

### Limitations

Our study represents a retrospectively conducted analysis. Exact timing from haemorrhage to treatment was only available in a subgroup of patients. The subgroup analysis however showed similar results. Due to the retrospective nature of this study, we were not able to analyse whether the delay in treatment for lower grade aSAH patients was due to initial misdiagnosis, suggested by previous studies, as this information was not collected^8–10^. This needs to be further evaluated. We however collected data on whether patients thought treatment directly at our location or were transferred from another hospital. The vast majority of patients with treatment delay were indeed transferred from another hospital. Another limitation of our study is that we did not collect information of specific treatment during the cerebral vasospasm (CVS) phase except for vasospasmolysis. In patients with delayed treatment we can therefore not make any conclusions whether complications were related or influenced by CVS treatment.

We did not analyse GOS on follow-up due to the amount of missing follow-up data. A sensitivity analysis of patients with available GOS on follow-up however did not show significant differences to GOS on discharge (data not shown).

## Conclusion

Timely treatment of ruptured IA is important to prevent complications and increase likelihood of favourable outcome. Treatment > 24 h of aneurysm rupture is associated with higher mortality and cerebral infarction rates, especially in lower grade aSAH. Delay in treatment primarily affects lower grade aSAH patients as they might present with only minor symptoms and even be misdiagnosed. If our findings are confirmed in a separate multicentre cohort, we would emphasise aneurysm occlusion within 24 h independently of the HH grade.

## Data Availability

The corresponding author can be contacted and anonymized data will be shared on request from any qualified investigator upon reasonable request.
